# The massive 340 megabase genome of *Anisogramma anomala*, a biotrophic ascomycete that causes eastern filbert blight of hazelnut

**DOI:** 10.1186/s12864-024-10198-1

**Published:** 2024-04-05

**Authors:** Alanna B. Cohen, Guohong Cai, Dana C. Price, Thomas J. Molnar, Ning Zhang, Bradley I. Hillman

**Affiliations:** 1 Department of Plant Biology, Rutgers The State University of New Jersey, New Brunswick, NJ 08901 USA; 2 Graduate Program in Microbial Biology, Rutgers The State University of New Jersey, New Brunswick, NJ 08901 USA; 3 Crop Production and Pest Control Research Unit, USDA-ARS, West Lafayette, IN 47907 USA; 4 Department of Botany and Plant Pathology, Purdue University, West Lafayette, IN 47907 USA; 5 Department of Entomology, Rutgers The State University of New Jersey, New Brunswick, NJ 08901 USA; 6 Center for Vector Biology, Rutgers The State University of New Jersey, New Brunswick, NJ 08901 USA; 7 Department of Biochemistry and Microbiology, Rutgers The State University of New Jersey, New Brunswick, NJ 08901 USA

**Keywords:** Ascomycete, Plant pathogen, Repeat-induced point mutation, Mating type gene, Transposon, Effector, *Anisogramma anomala*, Eastern filbert blight

## Abstract

**Background:**

The ascomycete fungus *Anisogramma anomala* causes Eastern Filbert Blight (EFB) on hazelnut (*Corylus* spp.) trees. It is a minor disease on its native host, the American hazelnut (*C. americana*), but is highly destructive on the commercially important European hazelnut (*C. avellana*). In North America, EFB has historically limited commercial production of hazelnut to west of the Rocky Mountains. *A. anomala* is an obligately biotrophic fungus that has not been grown in continuous culture, rendering its study challenging. There is a 15-month latency before symptoms appear on infected hazelnut trees, and only a sexual reproductive stage has been observed. Here we report the sequencing, annotation, and characterization of its genome.

**Results:**

The genome of *A. anomala* was assembled into 108 scaffolds totaling 342,498,352 nt with a GC content of 34.46%. Scaffold N50 was 33.3 Mb and L50 was 5. Nineteen scaffolds with lengths over 1 Mb constituted 99% of the assembly. Telomere sequences were identified on both ends of two scaffolds and on one end of another 10 scaffolds. Flow cytometry estimated the genome size of *A. anomala* at 370 Mb. The genome exhibits two-speed evolution, with 93% of the assembly as AT-rich regions (32.9% GC) and the other 7% as GC-rich (57.1% GC). The AT-rich regions consist predominantly of repeats with low gene content, while 90% of predicted protein coding genes were identified in GC-rich regions. Copia-like retrotransposons accounted for more than half of the genome. Evidence of repeat-induced point mutation (RIP) was identified throughout the AT-rich regions, and two copies of the *rid* gene and one of *dim-2*, the key genes in the RIP mutation pathway, were identified in the genome. Consistent with its homothallic sexual reproduction cycle, both MAT1-1 and MAT1-2 idiomorphs were found. We identified a large suite of genes likely involved in pathogenicity, including 614 carbohydrate active enzymes, 762 secreted proteins and 165 effectors.

**Conclusions:**

This study reveals the genomic structure, composition, and putative gene function of the important pathogen *A. anomala*. It provides insight into the molecular basis of the pathogen’s life cycle and a solid foundation for studying EFB.

**Supplementary Information:**

The online version contains supplementary material available at 10.1186/s12864-024-10198-1.

## Background

The investigation of biotrophic fungi – pathogens that require living host tissue – is complex and challenging. Because of their dependency on the host organism, biotrophs are difficult to isolate and grow in artificial media. They often have strict nutritional requirements and may require certain hormones or signaling chemicals secreted by the host to induce spore germination [1, 2]. Satisfying these conditions complicates any form of manipulation under laboratory conditions. Studies of rust fungi, which are basidiomycetes, and powdery mildew fungi, which are ascomycetes, highlight many of these challenges. Despite the significant economic impact of the resulting diseases, complete life cycles of these fungi have never been witnessed outside of their natural hosts. Consequently, despite substantial effort on the parts of many scientists, many details of host–pathogen interactions in rust and powdery mildew fungi remain poorly understood [3–5].

Advances in sequencing and bioinformatic tools have led to the rapid development of genomic techniques that facilitate investigation even of recalcitrant organisms. As the number of sequenced fungal genomes expands, patterns and features that are linked to obligate biotrophy have emerged [6, 7]. Genomic features, including both coding and non-coding elements, reveal characteristics of lifestyle and pathogen biology [8, 9]. A large repertoire of species-specific secreted small cysteine-rich proteins that represent candidate effectors is typical of biotrophs that have gene specific interactions with their host [10, 11]. Large genomes inflated with repetitive elements are another hallmark of biotrophic pathogens, as amplification of such elements contributes to a flexible genomic landscape that is highly adaptable to the gene-for-gene arms race that pathogens engage in with their hosts [11–13]. Identifying these characteristics of genomic features can fill in the blanks left by a lack of experimental data [14, 15].

One such fungal pathogen whose biology lacks understanding is *Anisogramma anomala,* an ascomycete within the order *Diaporthales*. *A. anomala* causes Eastern Filbert Blight (EFB), a devastating disease of hazelnut (*Corylus* spp.). The native host of *A. anomala,* American hazelnut (*C. americana*) tolerates infection, displaying mild disease symptoms and small, non-threatening cankers [16–18]. Both host and fungus are abundant on the east coast of the U.S. However, nearly all cultivars of the commercially important European hazelnut (*C. avellana*) are highly susceptible and develop severe perennial cankers that girdle stems, resulting in branch die-back and eventual tree death [19–21]. As such, EFB is the primary limiting factor of commercial hazelnut production in North America [22]. Historically, *C. avellana* cultivation was restricted to the Pacific Northwest region outside of the native range of *A. anomala*, limiting hazelnut cultivation to a fraction of its potential growth range [23]. Today, after an inadvertent introduction in the 1960s [24], EFB is widespread in the Pacific Northwest where it significantly impacts commercial production. Disease management costs were alleviated only recently by the release of resistant cultivars [25]. Despite the economic importance of *A. anomala* and considerable efforts now underway to breed for resistance [26], the EFB pathosystem remains poorly understood.

To support disease management and resistance breeding efforts, there is a need for a better understanding of the biology of *A. anomala* and the EFB pathosystem. However, *A. anomala* is an obligate biotroph, presenting many of the methodological difficulties as do rust fungi, powdery mildews, and other biotrophic pathogens [27]. The only useful source of tissue of *A. anomala* is ascospores extracted from the stromata of cankers of infected hazelnut, and successful subculture has not been achieved. Ascospores represent the only known spore stage of *A. anomala*; no conidial stage has been documented. Ascospores, by nature, are sexual spores and are not isogenic. While *A. anomala* ascospores can germinate and form small, branching germ hyphae, the fungus cannot be grown continuously in culture. It is predicted that *A. anomala* exhibits some form of self-inhibition, as ascospores will germinate in axenic culture only with the addition of an adsorbent such as activated charcoal or bovine serum albumin (BSA) [27]. Even with these additives, germinated ascospores exhibit poor growth and form small colonies (~ 0.25–0.5 mm in diameter) that yield little biomass [28]. Furthermore, the disease exhibits a complex, two-year infection cycle, which normally includes 15–18 months of latency, in which it is not feasible to visibly identity infected trees (Figure S1) [20, 21, 29–31].

Despite the challenges to performing experimental host/pathogen research, we saw the importance in understanding more about *A. anomala*, both as contributions to the U.S. hazelnut industry, and to plant pathogen biology. Due to the lack of an experimental system by which to study *A. anomala*, we used a genomic approach to elucidate features of EFB biology and pathogenesis. An earlier draft genome of *A. anomala* was assembled and mined for sequences that would be useful as simple sequence repeat (SSR) primers to examine population biology of the fungus and assist with resistance breeding [28]. That study revealed that the genome of *A. anomala* is surprisingly large, > 300 megabases (Mb) and consists of an abundance of transposons that constitute nearly 90% of the genome sequence. In this study, we present an updated and refined draft of the *A. anomala* genome sequence, its annotation, and analysis. Genomic analysis reveals characteristics of biotrophy, including a massive population of transposable elements (TEs), bimodal distribution of GC content, and a cache of genes encoding effector molecules. We also identified a number of genes that code for proteins predicted to be involved in pathogenesis and host/pathogen interactions. The annotated genome of *A. anomala* will serve as a vital resource for future research on the pathogen and EFB disease.

## Results

### *A. anomala* has a large, gene-poor genome

**Fig. 1:**
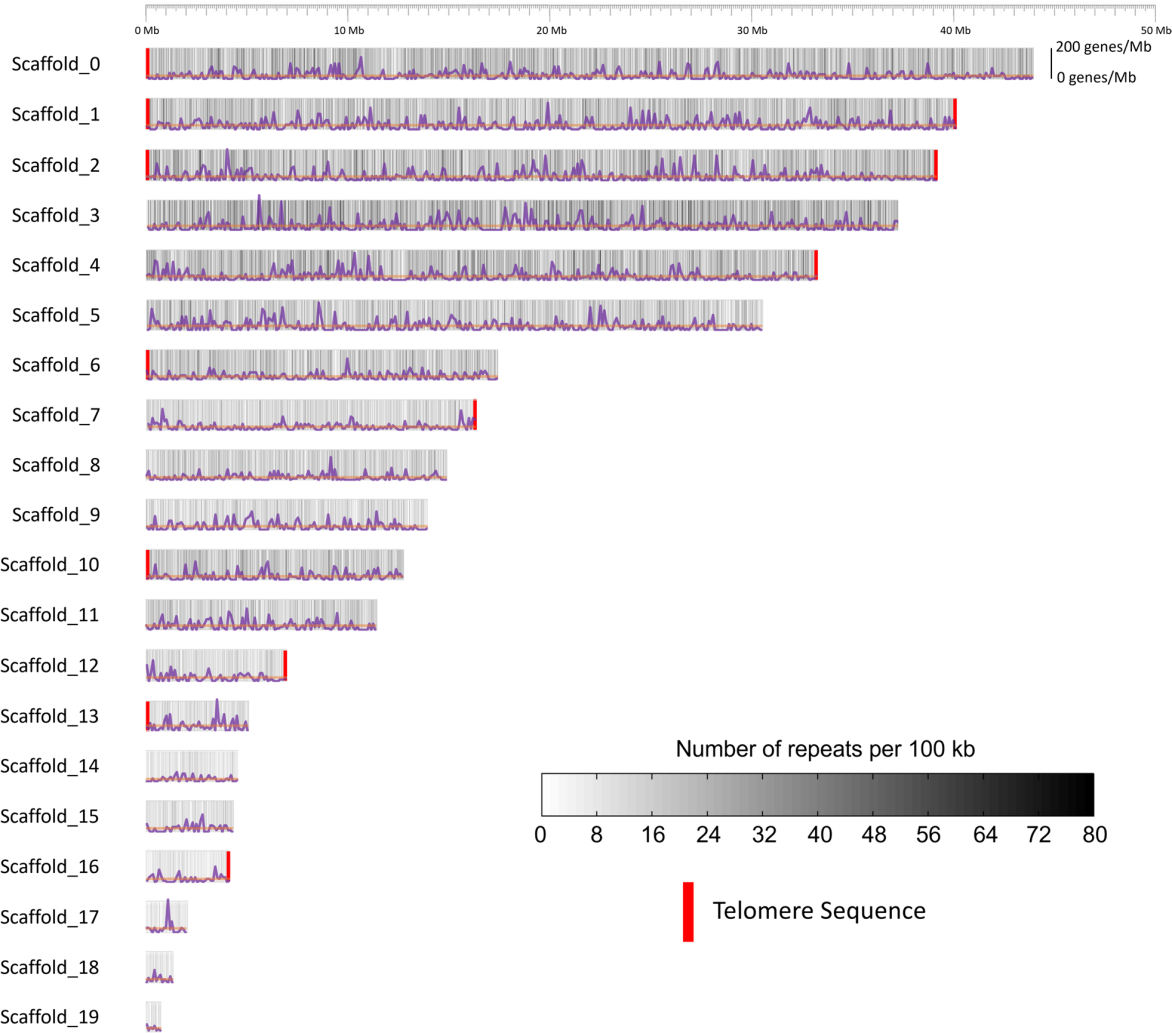
Repeat content and gene density distribution across major scaffolds (> 1Mb). Size of bar reflects the length of the scaffold (x-axis). Repeat density of dispersed repeats in bins of 100kb is represented as a heat map, ranging from white to black, the darker the color indicating higher repeat density. The height of each scaffold bar (y-axis) ranges from 0 to 200 genes/Mb with the average gene density of the scaffold plotted in orange. Gene density was calculated per 100kb and plotted along each scaffold in purple

The mate-pair and paired-end reads of genomic DNA for *Anisogramma anomala* OR1 generated over 31 Gb of data that were assembled into a 342,525,599 nucleotide (nt) genome with an average 91 × coverage (Table S1). The final assembly was distributed across 112 scaffolds with a GC content of 34.46%. Four scaffolds with a combined length of 27,247 nt were removed from further analysis as contamination, resulting in a final assembly size of 342,498,352 nt across 108 scaffolds. More than half of the assembly (N50) was on 5 scaffolds with an N50 scaffold length of 33.3 Mb. The largest scaffold was 43.9 Mb (Table 1). Nineteen major scaffolds (> 1 Mb) represent over 99% of the genome. This demonstrates a marked improvement over the first version of the assembly, published in 2013 [28] (Table S2). We identified telomere sequences (repeats of TTAGGG) on both ends of the second and third largest scaffolds with lengths of 40.1 and 39.2 Mb respectively, indicating these two scaffolds represent full-length chromosomes. Telomere sequences were also found on one end of 10 other scaffolds. Of the 19 largest scaffolds, telomere sequences were found on one end or both ends in 10 scaffolds (Fig. 1). On the contig level, the N50 was 196,655 bp and the L50 was 528 (Table 1).

**Table 1:** Table of assembly statistics and feature summary for the haploid genome of *A. anomala*

**Assembly Statistics**	
Scaffold Level	
Assembly size	342,498,352 bp
Total number	108
Largest	43,949,127 bp
N50	33,254,450 bp
L50	5
N99	1,308,775 bp
L99	19
# with telomere	12
% of gap region	0.37%
Contig Level	
Total number	3,310
N50	196,655 bp
L50	538
GC content	34.46%
**Annotatation Statistics**	
Protein coding genes	9,179
Average gene length	1,737 bp
Average protein length	481 aa
Mean exon number per gene	3.3
Mean exon length	438 bp
Eukaryotic BUSCOs	94.4%
Fungal BUSCOs	95.5%
Carbohydrate active enzymes	614
Glycoside hydrolases	298
Glycosyl transferases	154
Auxillary activities	83
Carbohydrate esterases	41
Carbohydrate-binding molecules	28
Polysaccharide lyase	10
Predicted secreted proteins	762
Candidate effectors	165

To evaluate the completeness of the *A. anomala* genome assembly, we performed flow cytometry using nuclei released from 8-week old mycelium. Based on flow cytometry, the genome size of *A. anomala* OR1 was estimated to be 370 Mb (Figure S2), slightly more than, but consistent with the genome assembly estimate.

Using a combination of RNA-seq evidence and ab initio gene prediction, we predicted 9,179 protein coding genes in the *A. anomala* genome. This gene set includes 94.4% of eukaryotic benchmarking universal single-copy orthologs (BUSCOs) and 95.5% of fungal BUSCOs. Average gene density on major scaffolds was approximately 25.8 genes/Mb and remained relatively consistent among major scaffolds (Fig. 1).

**Fig. 2:**
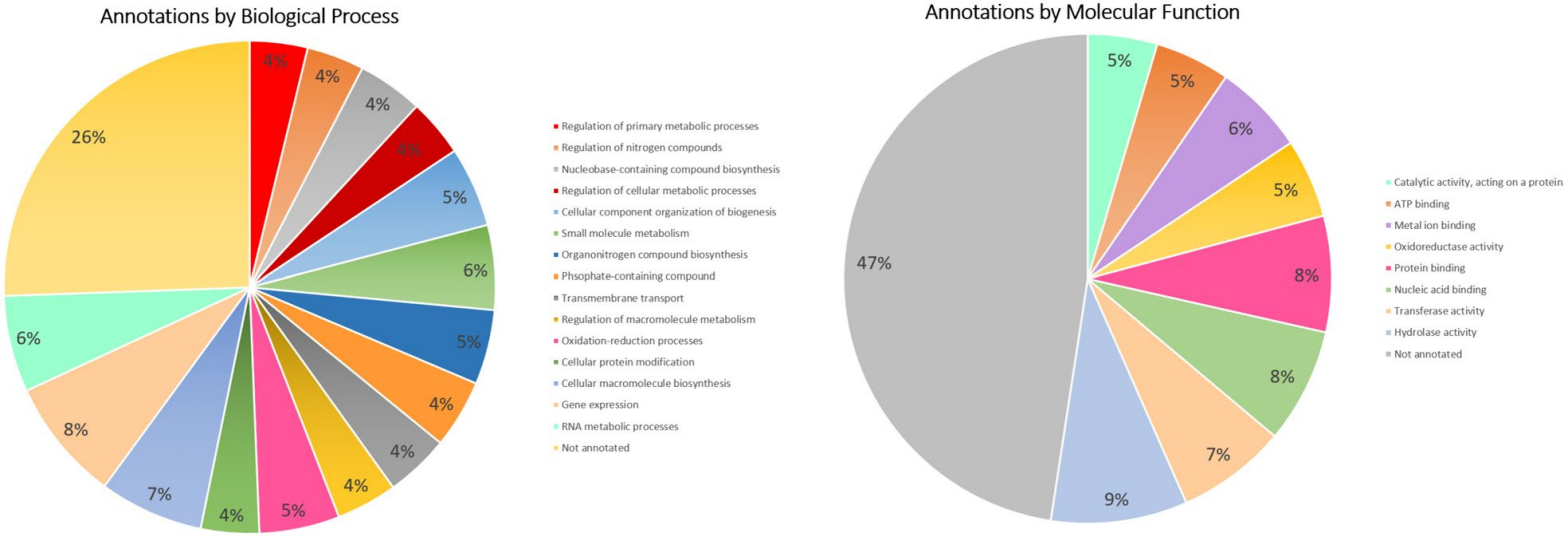
Annotation of *A. anomala* predicted gene models by Gene Ontology (GO) categories. Functions of genes are shown by biological process and molecular functions

Gene models were annotated with Gene Ontology (GO) terms merged with InterPro IDs. Eighty-eight percent of gene models had BLASTp hits against the NCBI nr database. Approximately 75% of gene models have been annotated by biological process and 50% with a molecular function (Fig. 2, Table S3). Gene models were also annotated with KEGG Orthology (KO) terms, using a combination of the KEGG Automated Annotation Server and BlastKOALA. Roughly 38% of protein sequences were assigned KO identifiers, which make up 99 complete or nearly complete KEGG pathways (Table S3).

### *A. anomala* has large arsenal of effectors and CAZymes

To identify proteins that may be involved in virulence and disease, we identified genes that code for potential effectors, molecules that are involved in host/pathogen interactions. We first identified 762 proteins with signal peptides as evidence of a secreted protein. Those proteins were then analyzed with EffectorP 2.0 to further predict potential effector proteins. One hundred and sixty-five proteins (1.8% of total proteins. 21.7% of secreted proteins) were predicted to be effector candidates (Table 1). All effector candidates were subjected to a BLASTp search of the NCBI nr database. Over half (55%) of candidate effectors returned no BLAST hit, and of those that did return a hit, 42% were hypothetical proteins or proteins with unknown function. For those effector candidates that match a protein with a known function, possible roles include one glycoside hydrolase, one cutinase, and two peptidases (Table S4).

**Fig. 3:**
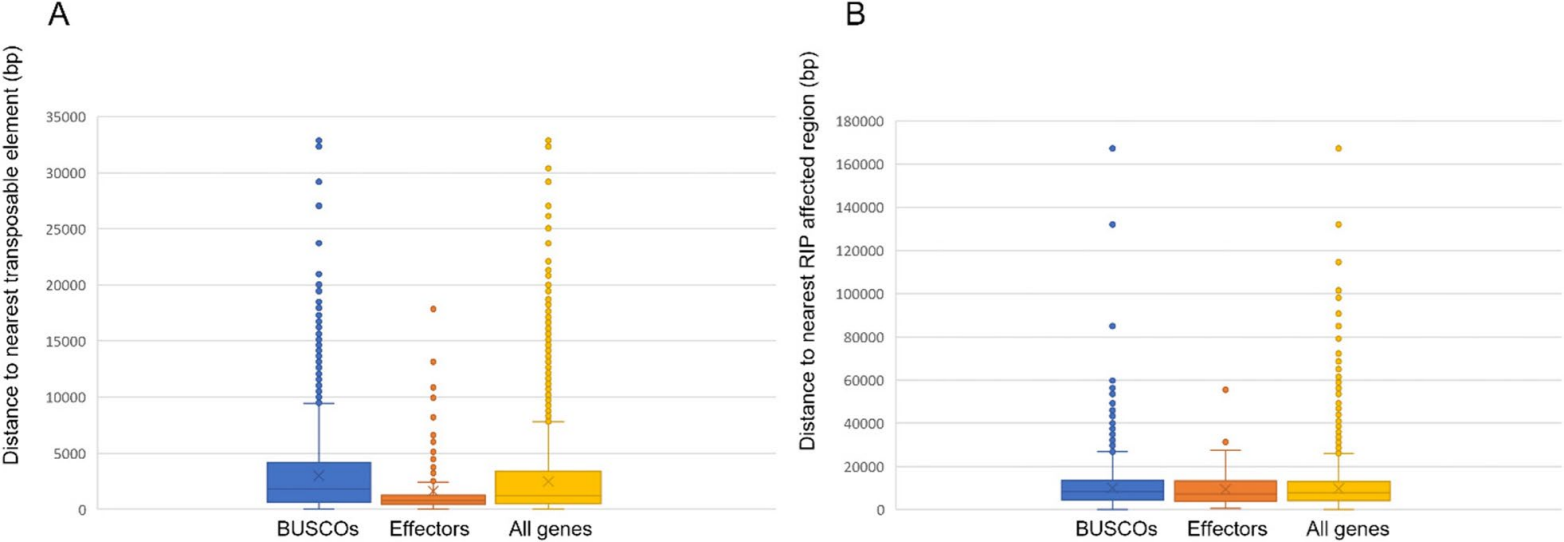
**A** Comparison of distance of well-conserved genes (BUSCOs), predicted effectors, and a randomized set of gene models from the nearest repeat element or **B** large RIP affected region (LRAR) as predicted by RIPPER software

Genes encoding putative effector molecules were evaluated for their proximity to the closest repeat element and the closest large RIP affected region (LRAR) as predicted by RIPPER. BUSCOs and a random subset of all genes were included for comparison (Fig. 3). On average, effectors were approximately 1.5 kb from the nearest TE while BUSCOs and a randomized set were 3 kb and 2.5 kb respectively. The closest distance to LRARs for effectors, BUSCOs, and the randomized set did not differ significantly from each other and averaged at 9900 bp, 9400 bp, and 9700 bp respectively.

In addition to effector molecules, we also identified carbohydrate active enzymes (CAZymes) that may play a role in plant pathogenesis. Using the dbCAN3 meta server, we identified 614 potential CAZymes. These proteins include 298 glycoside hydrolases, 154 glycosyl transferases, and 41 carbohydrate esterases (Table 1, Table S3). Finally, we identified biosynthetic gene clusters with the fungal version of antiSMASH. Twenty-five biosynthetic gene clusters were predicted, including 8 polyketide synthase (PKS), 7 terpene synthesis, 9 nonribosomal peptide synthetase (NRPS) clusters, and 1 PKS/NRPS combination cluster (Table S5).

Genome and annotation statistics including genome size, repeat content, and different categories of protein coding genes (effectors, CAZymes, and biosynethetic gene clusters) were compared to related fungi (Table 2). Like other biotrophic fungi, *A. anomala* has a large genome with high repeat content (shown below). A large number of effectors (relative to total protein coding genes), small number of biosynthetic gene clusters and CAZymes are other hallmarks shared between *A. anomala* and related biotrophic fungi.

**Table 2:** Genomic statistics of related fungi used for comparison in this study

	Accession	Lifestyle	Host	Disease	Genome Size	GC Content	Repeat Content	Encoded proteins	CAZymes	Percent CAZymes of total genes	Secreted proteins	Percent secreted proteins of total	Predicted Effectors	Percent Effectors of total	Biosynthetic Gene Clusters	Publication
Anisogramma anomala OR1	BioProject PRJNA966177	Obligate Biotroph	Hazelnut	Eastern Filbert Blight	342.5 Mb	34.46%	89.40%	9179	456	4.97	762	8.30	165	1.80	25	Current Study
Blumeria graminis f. sp. Tritici	GCA_905067625.1	Obligate Biotroph	Wheat	Powdery mildew	141.4 Mb	43.50%	75–90%	7,993	132	1.65	**970**	**12.14**	437	5.47	**9**	Wicker et al. 2013 [32]
Erysiphe neolycopersici	GCA_003610855.1	Obligate biotroph	Tomato	Powdery mildew	42 Mb	38%	39.40%	6851	135	1.97	499	7.28	175	2.55	3	Wu et al. 2018 [33]
Erysiphe pulchra	GCA_002918395.1	Obligate biotroph	Dogwood	Powdery mildew	63.5 Mb	38.50%	NA	6859	83	1.21	**341**	**4.97**	**70**	**1.02**	**7**	Wadl et al. 2019 [34]
Golovinomyces orontii	Golovinomyces orontii MGH1 v4.0	Obligate Biotroph	Wide range	Powdery mildew	211.35 Mb	42%	74.50%	12,649	**668**	**5.28**	157 (transcripts)	1.24	70 (transcripts)	0.55	**24**	JGI Project # 1,056,001; Micali et al. 2008 [35]
Melampsora larici populina	GCF_000204055.1	Obligate Biotroph	Poplar	Poplar leaf rust	101.1 Mb	41%	44.91%	16,257	308	1.89	1,796	11.05	1,184 (SSPs) **819**	5.04	**7**	Duplessis et al. 2011 [36]
Parauncinula polyspora	PRJEB29715 (ENA)	Obligate biotroph	Asian Oak	Powdery mildew	28.0 Mb	46.50%	8.50%	6046	104	1.72	261	4.32	70	1.16	**8**	Frantzeskakis et al. 2019 [37]
Puccinia graminis tritici	GCF_000149925.1	Obligate Biotroph	Wheat/cereals	Stem rust	88.6 Mb	43.30%	43.77%	15,979	203	1.27	1,386	8.67	1106 (SSPs)	6.92	**2**^a^	Broad Institute ASM14992v1
Ustilago maydis	GCF_000328475.2	Obligate Biotroph	Maize/cereals	Corn smut	19.7 Mb	54%	1.10%	6780	139	2.05	426	6.28	203	2.99	**13**	Kamper et al. 2006 [38]
Taphrina deformans	GCA_000312925.2	Biotroph	Peach/almond	Peach leaf curl	13.4 Mb	49.50%	1.50%	4663	231	4.95	**256**	**5.49**	24	0.51	4	Cisse et al. 2013 [39]
Fusarium graminearum	GCF_000240135.3	Hemibiotroph	Wheat/barley/maize	Fusarium head blight/ ear rot of corn	36.5 Mb	48%	0%	13,313	487	3.66	1442	10.83	190	1.43	67	Cuomo et al. 2007 [40]/King et al. 2015 [41]
Colletotrichum graminicola	GCF_000149035.1	Hemibiotroph	maize/cereals	Anthracnose leaf blight	51.6 Mb	49%	22.30%	12,019	572	4.76	1650	13.73	314	2.61	42	O'Connell et al. 2012 [42]
Pyricularia grisea	GCF_004355905.1	Hemibiotroph	Rice	Rice blast	44.6 Mb	47.50%	12.61%	12,452	539	4.33	1491	11.97	**490**	**3.94**	45	Gomez Luciano et al. 2019 [43]
Pyricularia oryzae	GCF_000002495.2	Hemibiotroph	Rice	Rice blast	41 Mb	51.50%	11.86%	12,989	538	4.14	752	5.79	370	2.85	30	Dean et al. 2005 [44]
Verticillium dahliae	GCA_000150675.2	Hemibiotroph	Wide range	Verticillium wilt	33.8 Mb	55.50%	5.00%	10,535	418	3.97	739	7.01	127	1.21	25	Klosterman et al. 2011 [45]
Botrytis cinerea	GCF_000143535.2	Necrotroph	wide range	Gray mold	42.6 Mb	41.50%	1%	11,698	1155	9.87	879	7.51	**130**	**1.11**	**24**^a^	Van Kan et al. 2017 [46]; Amselem et al. 2011 [47]
Cryphonectria parasitica	GCF_011745365.1	Necrotroph	Chestnut	Chestnut blight	43.9 Mb	50.80%	12.10%	11,606	548	4.72	397	3.42	32	0.28	46	Crouch et al. 2020 [48]
Diaporthe helianthi	GCA_001702395.2	Necrotroph	Sunflower	Stem canker	63.7 Mb	43.50%	NA	13,139	516	3.93	**1475**	**11.23**	**245**	**1.86**	67	Baroncelli et al. 2016 [49]
Sclerotinia sclerotiorum	GCA_001857865.1	Necrotroph	Wide range	White mold	38.9 Mb	41.50%	12.96%	11,130	411	3.69	603	5.42	57	0.51	15	Derbyshire et al. 2017 [50]
Valsa mali	GCA_000818155.1	Necrotroph	Apple	Apple canker	44.7 Mb	49%	14.10%	11,284	342	3.03	779	6.90	193	1.71	85	Yin et al. 2015 [51]
Nectria cocca	GCF_000151355.1	Saprotroph	Wide range	Root rot	51.3 Mb	50.50%	5.10%	15,708	889	5.66	**1379**	**8.78**	**257**	**1.64**	**38**	Coleman et al. 2009 [52]
Podospora anserina	GCF_000226545.1	Saprotroph	Non-phytopathic	NA	34.7 Mb	52%	5%	10,518	243	2.31	**267**	**2.54**	**1064**	**10.12**	31	Espagne et al. 2008 [52]
Stachybotrys chartarum	GCA_000730325.1	Saprotroph	Non-phytopathic	NA	36.9 Mb	53%	1.01%	11,530	383	3.32	**1206**	**10.46**	**144**	**1.25**	**55**	Semeiks et al. 2014 [53]
Neurospora crassa	GCA_000182925.2	Free-living	Non-phytopathic	NA	41 Mb	48%	10%	10,812	191	1.77	891	8.24	**167**	**1.54**	12	Galagan et al. 2003 [54]
Chaetomium globosum	GCF_000143365.1	Free-living	Non-phytopathic	NA	34.3 Mb	56%	6%	11,040	146	1.32	**1094**	**9.91**	177	1.60	**45**	Cuomo et al. 2015 [55]

Bold font indicates missing information from the respective study, numbers were predicted using the same methods used in the current study

^a^Indicates missing CDS data, Biosynthetic gene clusters were predicted using genomes only

### *A. anomala* genome hosts a large population of transposable elements (TEs)

The *A. anomala* genome hosts a large population of TEs that accounts for approximately 88% of the final genome assembly (Table 3). Repeat content remained relatively constant at 88% across major scaffolds (Fig. 1). The TE population consists of 2,536 individual repeat families, making up over 300,000 individual interspersed elements (Table 3). The vast majority (90%) of repetitive sequences was comprised of Long Terminal Repeat (LTR) retrotransposons, mostly *Copia*-like elements, which alone account for over half of the genome assembly. Eight of the ten repeat families with the highest copy numbers (> 7,000 members each) were identified as *Copia*-like elements.

**Table 3:** Detailed breakdown of the repeat population in the *A. anomala* genome. Repeat elements are classified by the name assigned by RepeatClassifier

	**Number of Elements**	**Length (bp)**	**Percent of Genome**
**Class I Elements**			
LTRs			
Copia-like	104,635	183,550,845	53.587
Gypsy-like	74,787	60,218,611	17.581
Other	68,679	27,882,387	8.14
LINEs			
L3/CR1	59	19,678	0.006
Tad1	4,623	5,848,916	1.708
Other	89	47,022	0.014
SINEs	136	370,136	0.108
**Class II Elements**			
Tc1-Mariner	47	6,543	0.002
CMC-Enspm	549	129,709	0.038
Helitron	3	3,705	0.001
**Unclassified**	51,711	28,213,842	8.237
**Total Interspersed Repeats**	305,318	306,291,394	89.42
**Simple Repeats**	19,952	937,149	0.27
**Low-Complexity**	1,402	77,435	0.02

### *A. anomala* exhibits “two-speed” genome

**Fig. 4:**
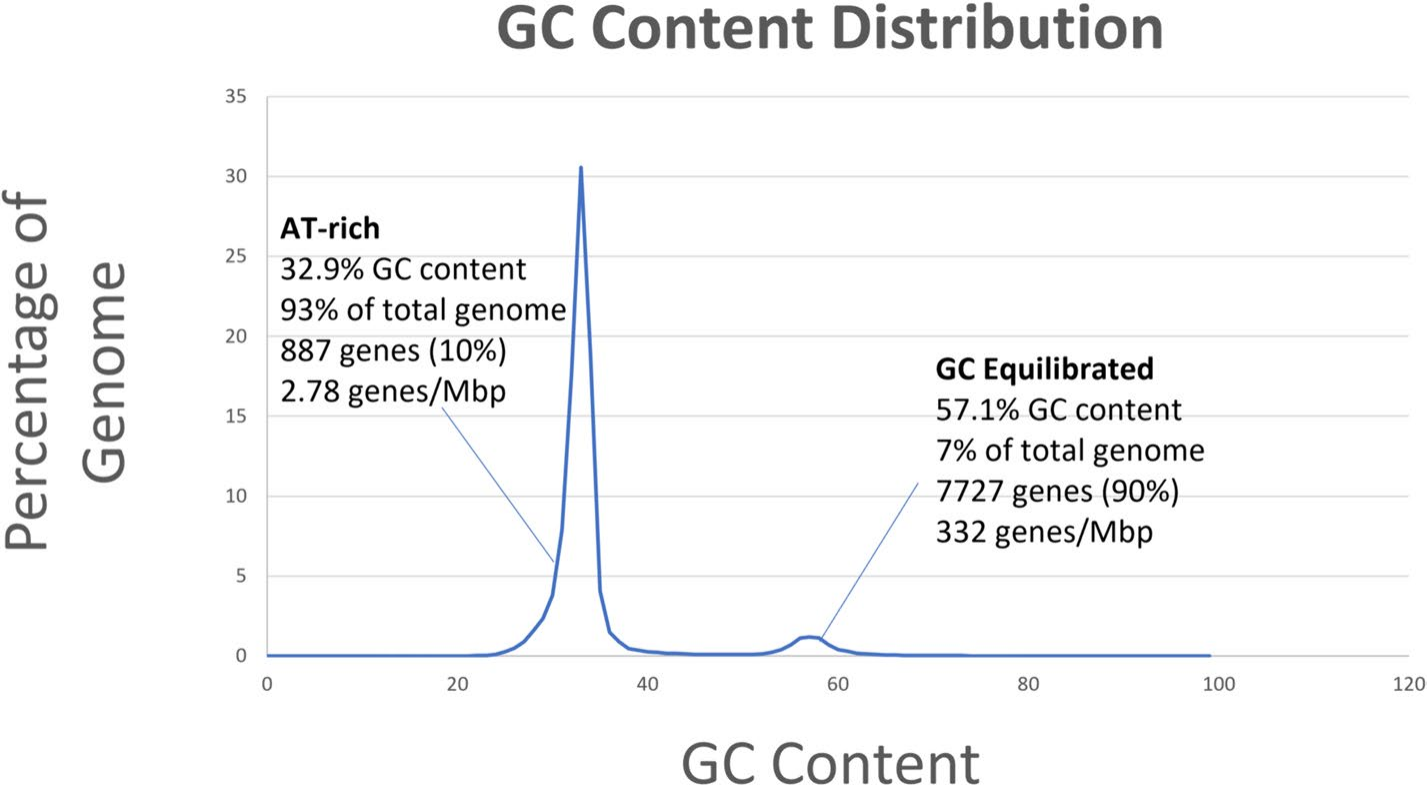
Distribution of GC-content across the *A. anomala* genome. The genome was broken up into regions using Jensen-Shannon divergence, for which GC-content was calculated. Proportions of the genome were assigned to GC-content in 1 percent increments

The overall distribution of GC-content across major scaffolds remained relatively constant at approximately 34%. However, measurement of proportions of GC-distribution across the entire genome reveals two peaks, indicating a bimodal genome (Fig. 4). The first peak, at 32.9% GC indicates AT-rich regions. This peak accounts for 93% of the genome and 10% (933/9,179) of the protein coding genes. These AT-rich regions are gene poor, with an average gene density of 2.93 genes/Mb. The second peak, at 57.1% GC indicates GC equilibrated regions that account for 7% of the genome and 90% (8,246/9,179) of protein coding genes. These GC-equilibrated regions are over 100-fold more gene-dense with an average of 344 genes/Mb.

We performed an enrichment analysis using the Fisher’s exact test of the gene models within AT-rich genomic regions (Table S6), sheet 1). A number of GO terms are over-represented (*p*-value < 0.05) including beta-glucan/cellulase metabolism, peptidase/hydrolase activity, and ion transport (Table 4). Additionally, despite these regions encoding only 10% of protein coding genes, 30% (49/165) of predicted effector coding genes were found in these AT-rich hotspots.

**Table 4:** GO term enrichment analysis of genes within the AT-rich regions of the *A. anomala* genome

GO ID	GO Name	*P*-value
GO:0051275	beta-glucan catabolic process	0.02887
GO:0051273	beta-glucan metabolic process	0.00894
GO:0032986	protein-DNA complex disassembly	0.01032
GO:0032984	protein-containing complex disassembly	0.04633
GO:0005381	iron ion transmembrane transporter activity	0.02887
GO:0031572	G2 DNA damage checkpoint	0.01032
GO:0030243	cellulose metabolic process	0.02887
GO:0030245	cellulose catabolic process	0.02887
GO:0007095	mitotic G2 DNA damage checkpoint	0.01032
GO:0016825	hydrolase activity	0.02677
GO:0004843	thiol-dependent protease activity	0.02679
GO:0034755	iron ion transmembrane transport	0.02887
GO:0006564	L-serine biosynthetic process	0.01032
GO:0008236	serine-type peptidase activity	0.02677
GO:0008233	peptidase activity	0.02319
GO:0017171	serine hydrolase activity	0.02677
GO:0031498	chromatin disassembly	0.01032
GO:0016702	oxidoreductase activity	0.03590
GO:0045727	positive regulation of translation	0.02887
GO:0072503	cellular divalent inorganic cation homeostasis	0.00894
GO:0072507	divalent inorganic cation homeostasis	0.00894
GO:0015940	pantothenate biosynthetic process	0.04633
GO:0015939	pantothenate metabolic process	0.04633
GO:0034250	positive regulation of cellular amide metabolic process	0.02887
GO:0070011	peptidase activity, acting on L-amino acid peptides	0.01257
GO:0006337	nucleosome disassembly	0.01032
GO:0006826	iron ion transport	0.02887
GO:0006874	cellular calcium ion homeostasis	0.00894
GO:0046915	transition metal ion transmembrane transporter activity	0.03967
GO:0004311	farnesyltranstransferase activity	0.01032
GO:1,902,653	secondary alcohol biosynthetic process	0.02887
GO:0018958	phenol-containing compound metabolic process	0.02887
GO:0051560	mitochondrial calcium ion homeostasis	0.01032
GO:0043022	ribosome binding	0.01349
GO:0055074	calcium ion homeostasis	0.00894

### *A. anomala* exhibits a number of unique gene families

**Fig. 5:**
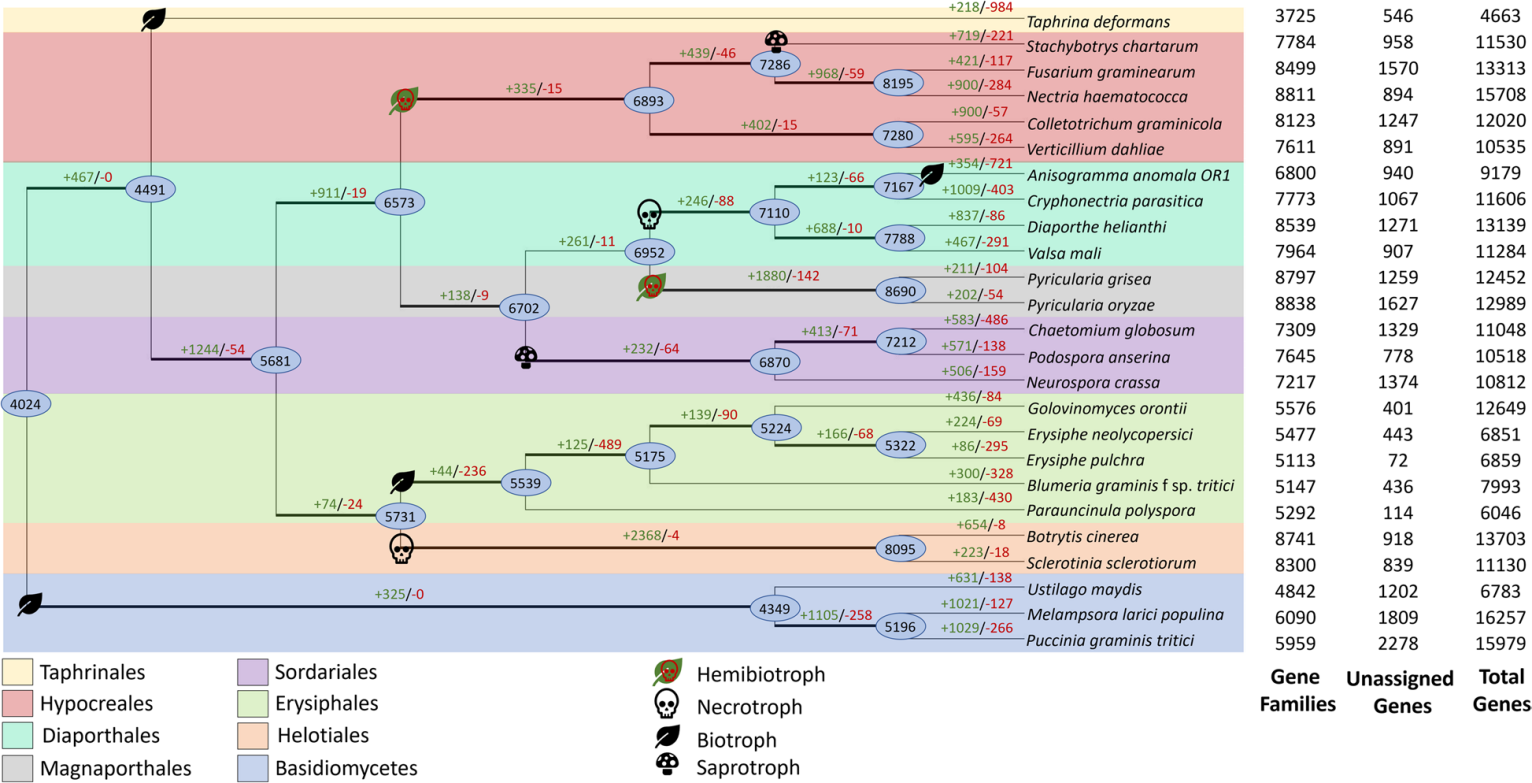
Predicted pattern of gene family gain and loss in representative fungal genomes. Cladogram representation of Maximum Likelihood phylogeny of *A. anomala* and 15 related fungi based on 2,800 single copy orthologues. The total number of protein families in each species or node is estimated by Wagner parsimony and stochastic mapping. The numbers of the branches correspond to gene family gain (green) or loss (red) and inferred ancestral protein families (in oval). The numbers of gene families, unassigned genes, and total gene numbers are indicated for each species

We performed an Orthofinder analysis to identify gene families shared with related fungal pathogens (Table S7). A super-gene phylogeny was constructed using 34 single-copy orthologous gene families and their corresponding protein sequences. Gene family counts were used to reconstruct ancestral gene family content and gain/loss of homologous gene families with Wagner parsimony and stochastic mapping (Fig. 5).

There are 1,121 gene models that are not identified as orthologous to related fungal pathogens and are likely specific to *A. anomala* (Table S6), sheet 2). Of these unique genes, 83 of them are predicted to code for effectors, indicating that over half of the predicted effectors are unique to *A. anomala*. GO terms overrepresented include beta-glucan and cellulose metabolism (*p*-value < 0.05), suggesting a role in production of plant degrading compounds (Table S8). An additional 450 GO terms are underrepresented, mostly including processes involved in central metabolism and fungal growth and development.

The Orthofinder analysis and Wagner parsimony revealed 354 genes families gained and 721 lost in *A. anomala* since diverging from its last common ancestor with *C. parasitica.* Gene families that are expanded or gained in *A. anomala* account for an additional 32 putative effector genes- meaning that approximately 70% of putative effectors are in species specific gene families or lineages of gene families that have expanded in *A. anomala.* GO terms overrepresented in gained/expanded gene families include catabolic processes and degradation of organic compounds (Table S9). The GO terms that are underrepresented include protein, organelle, and cellular biosynthetic processes.

### Transposable elements show evidence of Repeat-induced point mutation (RIP)

The *A. anomala* genome encodes two genes that exhibit sequence homology and are orthologous to *rid* (RIP defective) in *Neurospora crassa*. The two genes are predicted to encode a C5-DNA methyltransferase and a modification methylase respectively. Both genes have been assigned GO terms for methyltransferase activity. *A. anomala* also encodes a homolog of *dim-2*, an additional methyltransferase identified in *N. crassa* to be involved in the RIP process (Figure S3).

**Fig. 6:**
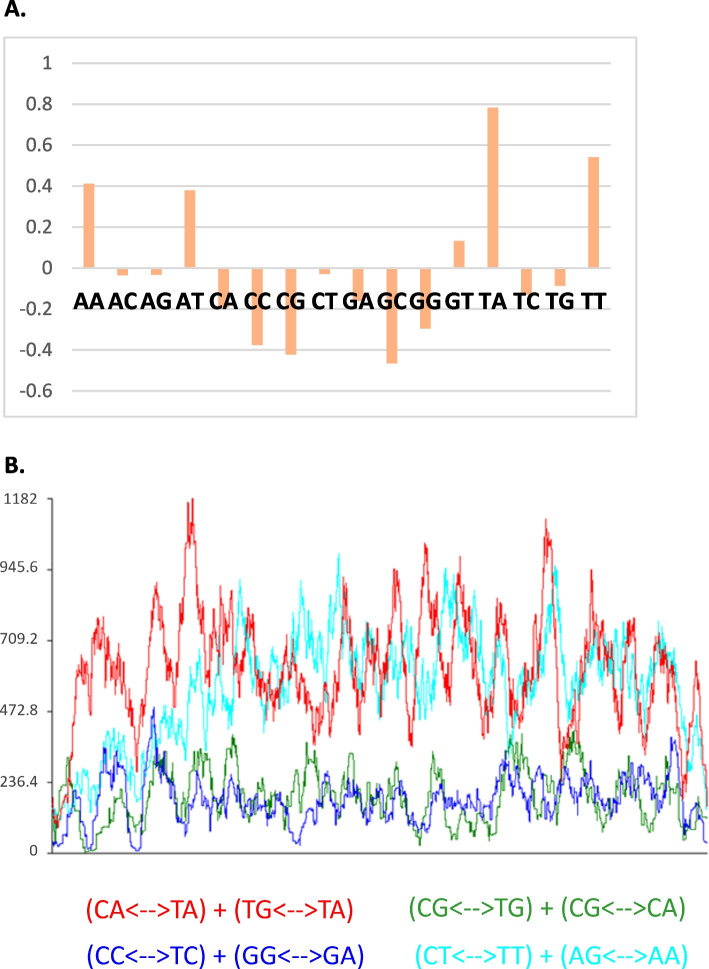
**a** Log(10) of average fold change in dinucleotide frequencies of all repeat families compared to non-repetitive control sequences. **b** Alignment based RIP analysis of repeat family with highest copy number (rnd-1_family-0). Each type of RIP mutation is represented by a different color, demonstrating the most dominant types of RIP within this repeat family

Dinucleotide frequencies and RIP indices were calculated for a subset of up to 100 members for all identified repeat families (Fig. 6a). Compared to a control of non-repeat sequences, repeat sequences exhibit an over-abundance of TpA (6.7 × more frequent) and TpT (4.1 × more frequent) dinucleotides and under-abundance of GpC (3.9 × less frequent) and CpG (2.8 × less frequent) dinucleotides. RIP indices were also calculated for the same subsets of repeat families (Table 5). The mean TpA/ApT index for repetitive sequences is 1.14, while non-repeat sequences have an index of 0.48. The mean (CpA + TpG)/(ApC + CpT) index is 0.087 in repetitive sequences and 0.095 in non-repetitive sequences. There were no significant differences in dinucleotide frequencies or RIP indices between repeat classes.

**Table 5:** Calculated RIP-indices for the five repeat families with the largest copy number. A TpA/ApT index ≥ 0.89 and (CpT + TpG)/(ApC + GpT) index ≤ 1.03 indicate RIP activity. The numbers presented are the mean values calculated for a subset of 100 repeat family members

Repeat Family	Classification	Copy Number	Mean TpA/ApT	Mean (CpA + TpG)/ (ApC + GpT)
rnd1_fam0	LTR-Copia	10,537	1.373688	0.6427065
rnd1_fam1	LTR-unknown	10,431	1.406927	0.644971
rnd1_fam2	LTR-Copia	8,315	1.658198	0.9923082
rnd1_fam3	LTR-Copia	7,814	1.306482	1.016627
rnd1_fam4	LTR-Copia	7,444	1.539685	0.944061

An alignment-based RIP analysis of the repeat family with the highest copy number shows that *A. anomala* exhibits two dominant kinds of RIP (Fig. 6b). CpA→ TpA and CpT→ TpT mutations were dominant over other RIP-like mutations. The top 10 repeat families with the highest copy number were also analyzed with the alignment-based RIP analysis and demonstrate the same RIP mutational preference.

### *A. anomala* demonstrates genetic basis for homothallism

**Fig. 7:**
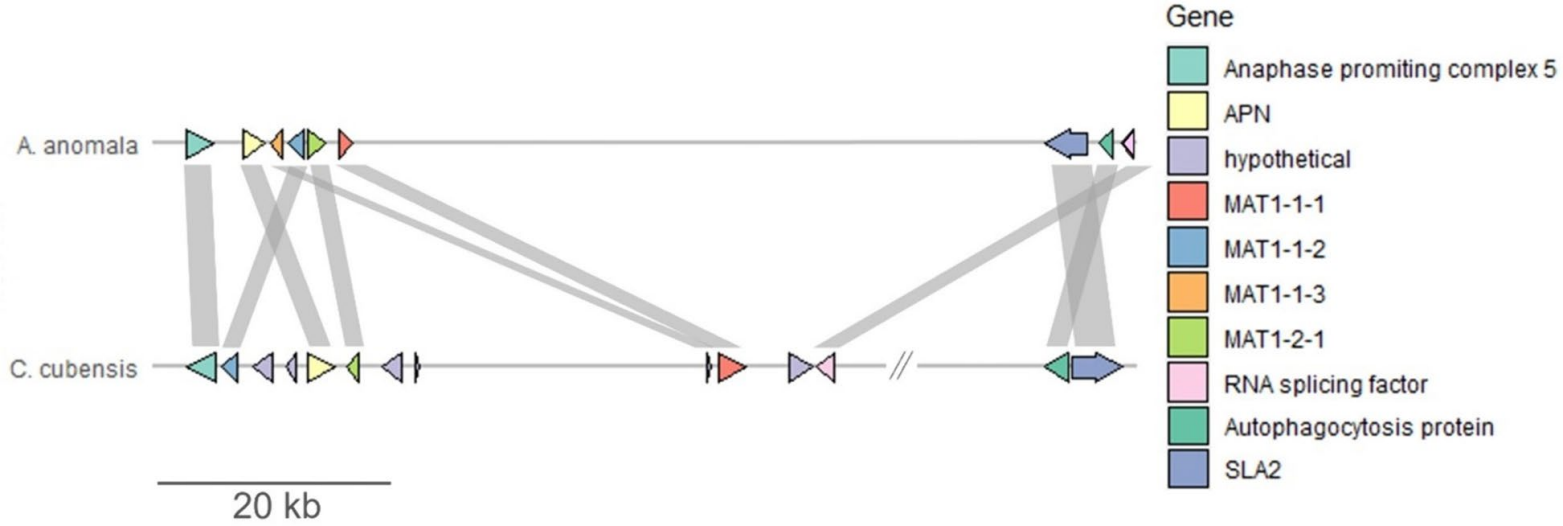
Genomic region corresponding to mating-type locus in *A. anomala.* Gene models were identified as MAT homologs through BLASTp search of NCBI database and analyzed for synteny compared to *Chrysoporthe cubensis,* a homothallic fungus in the *Cryphonectriaceae*

Homologs for both MAT1-1 and MAT1-2 idiomorphs have been identified in the *A. anomala* genome within the same 7 kilobase cluster (Table S6, sheet 3), consistent with evidence that the fungus is homothallic [20]. Homologs for the *mat* genes were identified through a BLASTp search of the NCBI nr database and verified by a pairwise sequence comparison to the corresponding genes in *Cryphonectria parasitica* [56]*.* Like the *C. parasitica* idiomorphs, three protein-coding genes are predicted to constitute MAT1-1 (MAT1-1–1, containing an alpha box motif; MAT1-1–2, a protein of unknown origin; and MAT1-1–3, containing an HMG motif), and a single protein-coding gene is predicted for MAT1-2 (MAT1-2–1, also containing an HMG motif). Within the *A. anomala* MAT locus, the gene encoding MAT1-2–1 was embedded between MAT1-1–1 and MAT1-1–2. Other genes usually associated with mating clusters in fungi, *apn2* and *sla2*, were identified in close proximity to the other MAT protein coding genes. The entire MAT cluster is largely syntenic to that of *Chrysoporthe cubensis,* a closely related homothallic fungus. The MAT loci of *A. anomala* is more compact and contains no additional genes besides those directly involved in determining mating type (Fig. 7). RNAseq data indicate that all four of these MAT genes were expressed constitutively (Figure S4).

## Discussion

The final genome assembly of *A. anomala* OR1 is approximately 343 Mb. This assembly is thought to be relatively complete based on genome size estimation compared to flow cytometry data as well as identified BUSCOs. The *A. anomala* genome is very large by fungal standards, almost 10 times the ~ 37 Mb size of the genome of the average ascomycete (Table 2) [32–35, 37–55, 57, 58]. However, large genomes are not uncommon amongst obligate biotrophic pathogens. Powdery mildew fungi, which are ascomycetes, have genomes in excess of 100 Mb [11], and rust fungi, which are basidiomycetes often with complex life cycles, may have genomes approaching 1 Gb [59]. Both of these unrelated fungi are subjected to the strong selective pressure imposed on biotrophic plant pathogens to maintain an intimate interaction with their host while avoiding recognition that initiates an immune response [60, 61]. The outcome of evolution driven by the pressure of a host/pathogen arms race is parallel adaptations resulting in remarkedly similar genomes among biotrophic pathogens [7, 11, 32–39].

The expansion of the *A. anomala* genome is driven by the proliferation of TEs, rather than accumulation of protein coding genes. The TE population is made primarily of LTR retrotransposons. *Copia*-like elements are by far the most abundant, which contrasts related fungi that are dominated by *Gypsy*-like repeats [48]. Despite the massive number of identified LTR retrotransposons, no single element has been determined to be intact with both 5’ and 3’ LTRs and the protein domains required for autonomous transposition, namely reverse transcriptase (RT), RNAse H (RH), and integrase (INT) [62]. One of the looming questions regarding the TEs in the *A. anomala* genome is how the invasion and uncontrolled replication of repetitive elements, largely of a single type of TE, was responsible for such extreme genome expansion.

Effector molecules play an important role in the colonization of biotrophic plant pathogens. Plants are able to recognize specific effector molecules through resistance (*R)* genes and activate a powerful hypersensitive response (HR) resulting in plant cell death which halts the spread of the invading pathogen [63]. If recognized, the pathogen is considered avirulent and the effector protein that triggers the HR response is characterized as an avirulence (*avr)* gene [64]. The pathogen responds by mutating or losing *avr* genes, so that they are no longer recognizable, or developing new effectors that avoid or suppress the effector-triggered immune response [65]. This relationship is the basis of the coevolutionary arms race between host plants and pathogens [66]. Most commercially available cultivars of *C. avellana* are protected by the R-gene “Gasaway”, named after the pollinizing cultivar that carried the dominant allele [67]. There is evidence that the Gasaway R-gene protects through an HR response [21]. However, Gasaway protected plants are overcome in regions of high pathogen pressure and diversity, suggesting that effectors and *avr* genes play a role in the breakdown of resistant cultivars [68–70].

For many putative effectors, there is no known function. As the goal is to be unrecognizable, there is no benefit to maintain conserved effector genes. However, we know that effectors can play multiple roles in establishing and maintaining infection [71, 72]. The large arsenal of putative effectors encoded in the *A. anomala* genome allows for flexibility. This is also why we have observed effectors in repeat-rich regions of genome, where there are high rates of mutation and recombination [73]. The compartmentalization of effectors and genes involved in pathogenicity in repeat rich regions fits the “two-speed” model of evolution [74].

CAZymes play an important role in both necrotrophic and biotrophic phytopathogenic infection. Necrotrophic fungi are known for having an arsenal of plant cell wall busting enzymes to launch an aggressive attack on their host. Necrotrophic ascomycetes code for between 600–800 CAZymes while *A. anomala* codes for 456 putative CAZymes, a typical number for biotrophic pathogens [75]. One of the most notable families of CAZymes encoded in the *A. anomala* genome is the glycoside hydrolase-18 (GH-18) family that includes all identified fungal chitinases [76]. It is predicted that both plant and fungal cell wall degrading enzymes are important for establishing biotrophic infection. Histological data of early infection of *A. anomala *on* C. avellana* shows a single germ hypha penetrating the plant cell wall, followed by the formation of intracellular vesicles [21]. CAZymes are required for initial penetration of the plant cell wall as well as the reformation of the fungal cell wall at the fungal/host interface [77]. Reasonable future steps would include investigating the expression of CAZymes during early infection to elucidate what genes are required to establish infection.

The Wagner parsimony analysis on Orthogroups of related fungal pathogens revealed the loss of 876 and gain of 285 gene families in *A. anomala* since it diverged from *C. parasitica.* Gene reduction in obligate parasites is a common trend, usually due to the loss of specific metabolic pathways as the parasite derives required compounds from their host [78]. KEGG pathway reconstruction revealed a number of missing or incomplete pathways for the biosynthesis of several amino acids including lysine, tryptophan, and asparagine [79]. It should be noted that the culture medium used for *A. anomala* contains yeast extract as well as additional asparagine to encourage growth [27]. Genes involved in pathways involved in energy generation (NADH dehydrogenase, nitrate reduction/assimilation) are missing as well. *A. anomala* exhibits parallel evolution to unrelated obligate biotrophic fungal pathogens that have independently lost similar biosynthetic and metabolic pathways [60, 80, 81].

The exception to the trend of gene loss is genes or gene families that encode effectors. Gene families that are unique or expanded in *A. anomala* contain 70% of predicted effectors. Biotrophs use effectors to maintain an intimate signaling relationship during infection [82, 83]. The need for a large and diverse effector arsenal drives the evolution of effector diversification and expansion [84, 85] as we observed with *A. anomala*. GO terms overrepresented in unique or expanding families include transmembrane transporters that are involved in the secretion of secondary metabolites that participate in pathogenesis. Amylases, peptidases, and catabolic activity GO terms are overrepresented in expanded families, likely aiding in adaptation to the obligate biotrophic lifestyle [10].

Despite TEs accounting for 88% of the final genome assembly, very few of these elements contain intact protein domains required for autonomous transposition. Sequences from all identified repeat families show evidence of RIP mutation. RIP is a defense mechanism that protects fungal genomes from TEs expanding unchecked [86, 87]. RIP functions by recognizing stretches over 400 bp of DNA with high (> 80%) sequence identity. The DMNT-1 homologue RID (RIP defective) methylates cytosine residues, which then undergo spontaneous deamination into thymine. This induces C→ T and G→ A transitions in both copies of duplicated sequences, resulting in permanent mutational changes in the DNA sequence [88, 89].

Fungi that demonstrate evidence of RIP vary in the degree or effectiveness by which RIP acts on the genome. *Neurospora crassa,* in which RIP was first described [90], has a very efficient RIP system, to the point where *N. crassa* has almost no duplicated sequences, TEs nor duplicated genes [91]. In other fungi, RIP is often demonstrable, but less effective [92]. In the related fungus *C. parasitica*, roughly 14% of the 43.9 Mb genome represented TEs, and there was some limited evidence for RIP [48, 92]. The *A. anomala* genome exhibits indications of RIP activity. The RIP indices calculated for repeat families exceed the accepted threshold for RIP activity (TpA/ApT ≥ 0.89 and (CpT + ApT)/(ApC + CpT) ≤ 1.03) [93, 94] and the dinucleotide frequencies demonstrate a depletion of pre-RIP dinucleotides and an enrichment of post-RIP dinucleotides in repeat regions compared to non-repeat regions [92]. In spite of evidence of a functional RIP pathway, transposons have managed to overtake the *A. anomala* genome. The massive expansion of the TE population in the *A. anomala* genome underscores the observation that mere presence of an apparently functional RIP system is no guarantee that TEs will be held in check.

In addition to defending against the uncontrolled replication of TEs in a genome, RIP is a major driver of genome evolution. RIP induces mutations on duplicated sequences, but those mutations often bleed into neighboring regions, so called “leaky RIP” [86, 95, 96]. Furthermore, the G/C→ T/A mutations have a major impact on GC content of a genome. The GC content of the *A. anomala* genome is relatively low, at 34%, however, it is not equally distributed across the genome. GC-proportion distribution reveals two peaks; 93% of the genome landscape has a GC-content of 32%. These stretches of GC-poor containing DNA are broken up by GC-rich blocks that are gene-rich and TE-poor. These data demonstrate that *A. anomala* fits the “two-speed” genome model [96–98].

Analysis of the mating-type locus revealed that *A. anomala* has the genes for both *MAT1-1* and *MAT1-2* idiomorphs, providing molecular evidence to support the previous evidence for homothallism [20]. Mating type systems, processes, and their associated genes are extraordinarily complicated in fungi, and many genes other than the MAT genes themselves may have different roles in the reproductive process [99]. In addition to controlling sexual development, MAT genes may be important in growth and virulence, including regulation of secondary metabolites and hyphal morphology.

Homothallism, such as that in *A. anomala*, is thought to be an evolutionary destination from which there is no likely return to a progenitor heterothallic state, an idea that was supported through research with *Neurospora* shifting multiple times from heterothallic to homothallic lifestyle, but never the reverse [100]. *Chrysoporthe* which is closely related to *Anisogramma,* has MAT locus features similar to *Neurospora*, including pronounced influence of retrotransposons, but there was some evidence to suggest that the *MAT1-2* and *MAT1-1* idiomorphs of the heterothallic *C. austroafricana* evolved from a homothallic progenitor [101]. In both the case of *Neurospora* and *Chyrosporthe,* the evolutionary transition of mating type is facilitated by TEs within the *mat* locus. Like the rest of the *A. anomala* genome, the *mat* locus is flanked by TEs. But the core genes for each idiomorph are found within the same 7 kb block with no TEs or additional genes. The mating cluster of *C. cubensis* includes additional genes not found in *A. anomala* as well as a 200 kb insertion of DNA that contains over 60 genes not related to determination of mating type (Fig. 7). One of the roles of sex in fungi and other organisms is to bring genetic variation to the species. It seems that a combination of homothallic sex and rampant genome invasion and expansion by transposons brings sufficient variability to *A. anomala*.

## Conclusions

At nearly 350 Mb, the *A. anomala* genome represents the largest ascomycete genome yet characterized. Gene number and putative functions are typical of fungal plant pathogens, but runaway amplification of repeat sequences has led to a massively bloated genome, despite hallmarks of functional genome surveillance by RIP. The *A. anomala* genome characterization will serve as a resource for others investigating this economically important plant pathogen, and for those interested in fungal genome evolution.

## Methods

### Fungal strain

*A. anomala* is an obligate biotroph that has not been grown in continuous culture, so tissue is scarce and not clonal. Based on knowledge of EFB epidemiology [102, 103], the *A. anomala* population in Oregon is believed to be decedents of a single introduction event from east of the Rocky Mountains and belong to a single lineage. But mycelium from different trees in fields is not clonal, and DNA or RNA extracted from a collection of germinated ascospores is also not clonal. The closest approximation we have to homogeneous tissue is to harvest ascospores from a single canker on a single tree, with the understanding that it most likely represents the result of a single infection. We collected ascospores from individual cankers from infected branches harvested from hazelnut plants growing at the Oregon State University Smith Horticultural Research Farm, Corvallis, OR. These plants had been inoculated 18 months prior in the greenhouse using local diseased plant material as inoculum source. We designate the strain presented here Oregon1, OR1.

Ascospores were extracted following the protocol we used previously [28]. Briefly, the branches were cut into pieces 5–7 cm in length, and surface-sterilized for 3 min in 10% bleach (0.525% sodium hypochlorite) followed by 1 min in 70% ethanol. After rinsing with sterile H_2_O, the stromata were hydrated in sterile H_2_O for 30 min and air-dried. The top of a canker was cut off with a sterile razor blade to expose the necks of the perithecia, and another sterile razor blade was inserted under the perithecia to provide pressure from below and push ascospores out of perithecial neck. The spores from individual cankers were suspend in sterile H_2_O containing 10 ppm rifampicin and 100 ppm streptomycin and quantified with a hemocytometer. We found one canker that produced approximately 5.5 M ascospores and these spores were used in this study unless noted otherwise.

To generate primary mycelium, a portion of the ascospores was adjusted to 1 × 10^5^ spores per ml and used to inoculate plates of culture medium overlaid with cellophane. The rest of the ascospores were stored at -80 °C. Half a milliliter of the spore suspension was spread on the cellophane surface in individual 9-cm diameter petri dishes. The medium contained (per liter) 2.7 g modified Murashige and Skoog basal salt mixture; 20 g sucrose; 2 g yeast extract; 2 g L-Asparagine; 15 g Bacto agar; 0.25 g activated charcoal; and 10 mg Rifampicin [27]. The cultures were grown at 18 °C in the dark for 8 weeks, by which time many spores had germinated and grown into opaque, whitish colonies approximately 0.25–0.5 mm in diameter. Mycelium was harvested by rinsing the cellophane with sterile H_2_O. A subset of plates was kept for four more weeks. By then the small colonies were turning grey and black, and the senescent mycelium was harvested as described above.

### Nucleic acid extraction, genome sequencing and assembly

Mycelium from 8-week-old cultures were used for DNA extraction using Gentra Puregene kit (Qiagen) following the fungi protocol. One paired-end DNA library with insert size approximately 350 bp (excluding adapters) was constructed using the TruSeq DNA Sample Prep kit (Illumina). Three mate-pair DNA libraries with insert sizes approximately 3 kb, 6 kb, and 10 kb, respectively, were constructed using the Nextera Mate Pair Library Prep kit (Illumina) following manufacturer’s instructions. All libraries were sequenced on the Illumina MiSeq platform.

The paired-end reads were trimmed with Trimmomatic v0.32 [104] in paired-end mode to remove adapter sequences and reads shorter than 100 bp after trimming were dropped. The mate-pair reads were first trimmed with Trimmomatic in paired-end mode to remove external adapters, then trimmed with Trimmomatic in single-end mode to remove internal adapters at ligation junctions. Reads shorter than 35 bp after trimming were dropped. The resulting reads were processed with a custom Perl script and only read pairs meeting the following conditions were retained for genome assembly: 1) both reads must have survived adapter trimming; 2) for read pairs in which external adapters were found, the junction adapter must be found in both reads; 3) for read pairs in which external adapters were not found, junction adapter must be found in at least one read. After data processing, the sequence reads were assembled using AllPaths-LG release 52,155 with default settings. [105]. Assembled scaffolds were subjected to a BLASTn search of the GenBank database release 258 [106]. Any scaffolds where the top hit was not fungal were removed as contamination.

### Flow cytometry

One hundred micrograms of freshly harvested 8-week old mycelium were cut into fine pieces with a sterile razor blade in 500 μl LB01 buffer on ice to release the nuclei [107]. The mixture was passed through a 40 μm filter and washed with 200 μl LB01 buffer. Nuclei from 50 mg young radish leaf, which has a 2C genome size of 1.1 Gb, were released the same way and used as control. Nuclei solutions were treated with RNase A and stained with propidium Iodide at room temperature for 20 min in darkness and run through a Beckman Cytoflex flow cytometer. The experiment was repeated three times.

### Repeat identification and masking

The assembled genome was soft-masked prior to gene prediction [108]. A comprehensive, non-redundant repeat library was created by integrating output from RepeatModeler [109, 110], TransposonPSI [111], and LTRharvest [112]. RepeatModeler v1.0.11 and TransposonPSI were run using default parameters to generate the first two repeat libraries. The third repeat library was built using LTRharvest. False positives were removed from the LTRharvest library by running LTRdigest with protein HMMs from Pfam [113] and GyDB [114] databases. LTR retrotransposons without domain hits were removed from the LTRharvest repeat library.

Each of the three repeat libraries was classified using RepeatClassifier, part of the RepeatModeler program suite, with Repbase version 23.08 [115], for consistency in identification and naming of repeat elements. The three repeat libraries were then merged and clustered with CD-HIT [116] at ≥ 80% identity to create a non-redundant library [117]. This custom library was used to soft-mask the *A. anomala* genome using RepeatMasker with the “xsmall” argument and default parameters [118].

### Transcriptome sequencing, gene prediction and annotation

Ascospores, 8-week old mycelium and 12-week old senescent mycelium were used for RNA extraction using the Plant RNeasy kit (Qiagen) following manufacturer’s instructions. Three mRNA libraries, one for each sample, were prepared using the TruSeq RNA Preparation kit (Illumina) following manufacturer’s instructions. The libraries were sequenced on the Illumina MiSeq platform.

Gene models were predicted using the BRAKER2 annotation pipeline [119], incorporating GeneMark-ET [120, 121] and Augustus [122] for ab initio and evidence-based gene prediction. RNA-Seq reads were mapped to the genome assembly using STAR [123]. The RNA-Seq mapping results were used as evidence for gene prediction in the BRAKER2 pipeline, using the “fungus” argument for fungal gene prediction. Genome completeness was assessed through a BUSCO analysis of benchmarking eukaryotic and fungal single-copy orthologs [124].

Blast2GO v5.2.5 [125] was used to perform a BLASTp search of the NCBI nr database with E-value cutoff of 1e-3. Interproscan v5.53 [126] results were imported in to Blast2GO and merged with GO annotations. KEGG annotation terms [79, 127] were assigned using a combination of BlastKOALA v2.2 [128] and the KEGG Automatic Annotation Server (KAAS) [129] searched against eukaryote and prokaryote KEGG GENES databases (release v89.1), with the single-directional best hit method.

Secreted proteins were predicted using SignalP 5.0 [130] to identify signal peptides sequences. Predicted secreted proteins were then analyzed with EffectorP 2.0 [131, 132] to predict genes encoding for potential effectors. Evidence including protein size and cysteine content was used for effector prediction. Potential function of effectors was evaluated by a BLASTp search [133] of the GenBank nr database (release 239) [106] with an e-value cutoff of 0.001. Functional domains were assigned using CD-Search webserver with default settings against the Conserved Domain Database v3.20 [134, 135]. Carbohydrate active enzymes were predicted using the dbCAN3 meta server [136–138] which integrates HMMER [139], DIAMOND [140], and Hotpep [141] searches of the CAZy database [142]. Biosynthetic gene clusters were predicted and identified using antiSMASH v5.1.2 [143].

### GC-content distribution

Analysis of GC-content was performed by segmenting genomic sequences into regions of differing GC-content using the Jensen-Shannon divergence at each sequence position calculated using OcculterCut v1.1 [144]. Gene models associated with AT-rich genomic regions were used as a test set in a GO term enrichment analysis test using a two-tailed Fisher’s Exact Test with a filter value of 0.05 with BLAST2GO v5.2.5 [125].

### Fungal super-gene phylogeny

We collected proteomes from 24 related ascomycete species to identify orthologous gene families. OrthoFinder v2.2.6 [145] was used under default settings to build orthogroups. Thirty-four single-copy orthologous gene families and their corresponding protein sequences were retrieved and aligned with MUSCLE v3.8.31 [146] and alignments were trimmed with TrimAl v1.4 [147] using the automated feature to select the best method. The trimmed alignments were automatically concatenated and partitioned using IQ-TREE v1.7-beta17 [148, 149]. The maximum likelihood tree was reconstructed with IQ-TREE under the LG + I + G model as selected using ModelFinder [150].

Gene family counts from the Orthofinder analysis were used to reconstruct ancestral gene family content and gain/loss of homologous gene families. These traits were reconstructed using Wagner parsimony in the Count software package [151] as well as stochastic mapping with GLOOME [152].

### RIP analysis

RIP indices of individual repeat copies were calculated in RStudio v1.1.414 [153] using a custom R (v4.1.2) script (file S1) and the Biostrings package v2.62.0 [154]. RIPCAL v2 [155] was used for alignment based analysis of repeat families and calculations of mutation frequencies. Large RIP affected regions (LRARs) were identified by a minimum of seven consecutive sliding windows (window size = 1000 bp, slide size = 500 bp) with a minimum RIP product value of 1.1, maximum RIP substrate value of 0.75 and minimum composite (product – substrate) value of 0.01. RIP product, substrate, and composite values and LRAR analysis was performed using The RIPper [156].

### Supplementary Information

**Supplementary Material 1.**

**Supplementary Material 2.**

**Supplementary Material 3.**

**Supplementary Material 4.**

**Supplementary Material 5.**

**Supplementary Material 6.**

**Supplementary Material 7.**

**Supplementary Material 8.**

**Supplementary Material 9.**

**Supplementary Material 10.**

**Supplementary Material 11.**

**Supplementary Material 12.**

**Supplementary Material 13.**

**Supplementary Material 14.**

## Data Availability

The *Anisogramma anomala* OR1 genome sequence and assembly have been submitted to NCBI SRA and have been given the BioProject reference number PRJNA966177. Protein and coding sequence fasta files can be found on FigShare under identifiers 24,905,166.v1 and 24,898,656.v1 respectively.

## Abbreviations

BUSCO: Benchmarking single-copy orthologs
CAZymes: Carbohydrate-active enzymes
EFB: Eastern Filbert Blight
GO: Gene ontology
KEGG: Kyoto encyclopedia of genes and genomes
LRAR: Large RIP-affected region
RIP: Repeat-induced point mutation
TEs: Transposable elements
