# Supplementary material for: The massive 340 megabase genome of Anisogramma anomala, a biotrophic ascomycete that causes eastern filbert blight of hazelnut
Source: BMC Genomics. 2024 Apr 5;25:347. doi: 10.1186/s12864-024-10198-1 (PMC10998396; doi:10.1186/s12864-024-10198-1)
Supplement: Supplementary file 1 — Supplementary Material 1. [file 12864_2024_10198_MOESM1_ESM.docx]

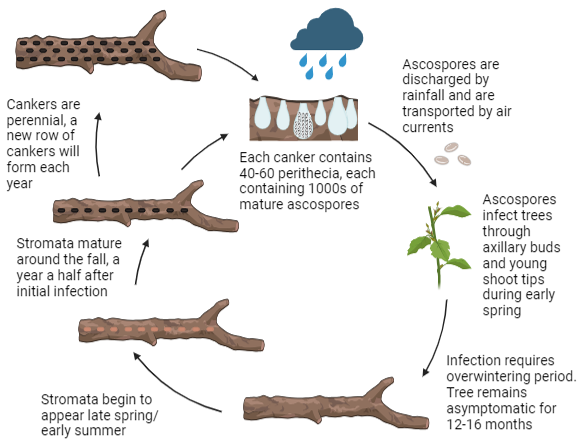


**Figure S1:** Infection cycle of *Anisogramma anomala* causing Eastern Filbert Blight on *Corylus* spp.
