# Supplementary material for: The massive 340 megabase genome of Anisogramma anomala, a biotrophic ascomycete that causes eastern filbert blight of hazelnut
Source: BMC Genomics. 2024 Apr 5;25:347. doi: 10.1186/s12864-024-10198-1 (PMC10998396; doi:10.1186/s12864-024-10198-1)
Supplement: Supplementary file 4 — Supplementary Material 4. [file 12864_2024_10198_MOESM4_ESM.docx]

**
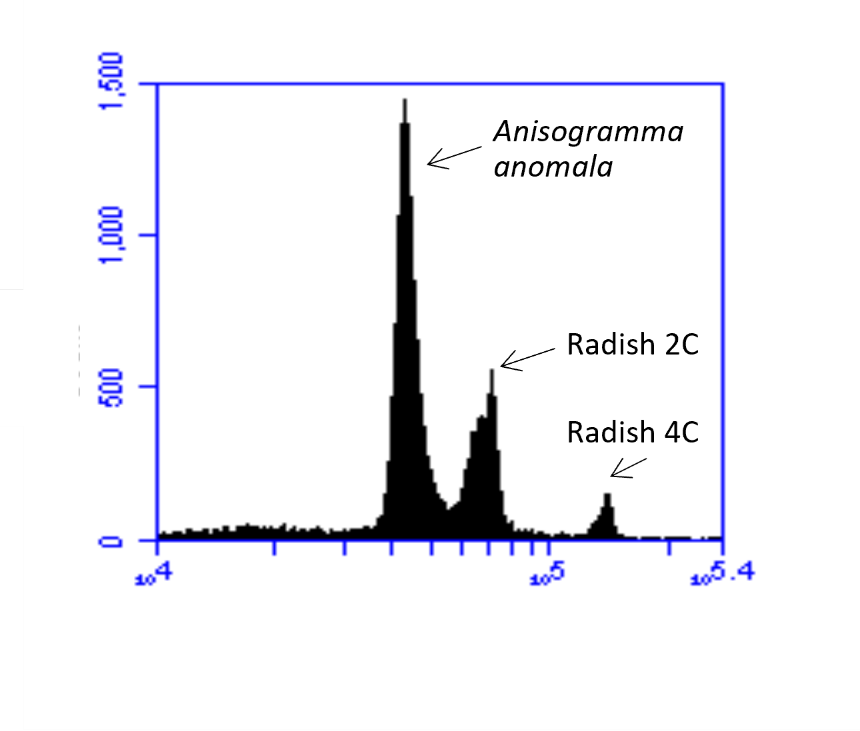
**

**Figure S2:** Determination of the genome size of *A. anomala* using flow cytometry. Radish, which has a 2C genome size of 1.1 Gb, was used as control
