# Supplementary figures and images for: The massive 340 megabase genome of Anisogramma anomala, a biotrophic ascomycete that causes eastern filbert blight of hazelnut
Source: BMC Genomics. 2024 Apr 5;25:347. doi: 10.1186/s12864-024-10198-1 (PMC10998396; doi:10.1186/s12864-024-10198-1)

**
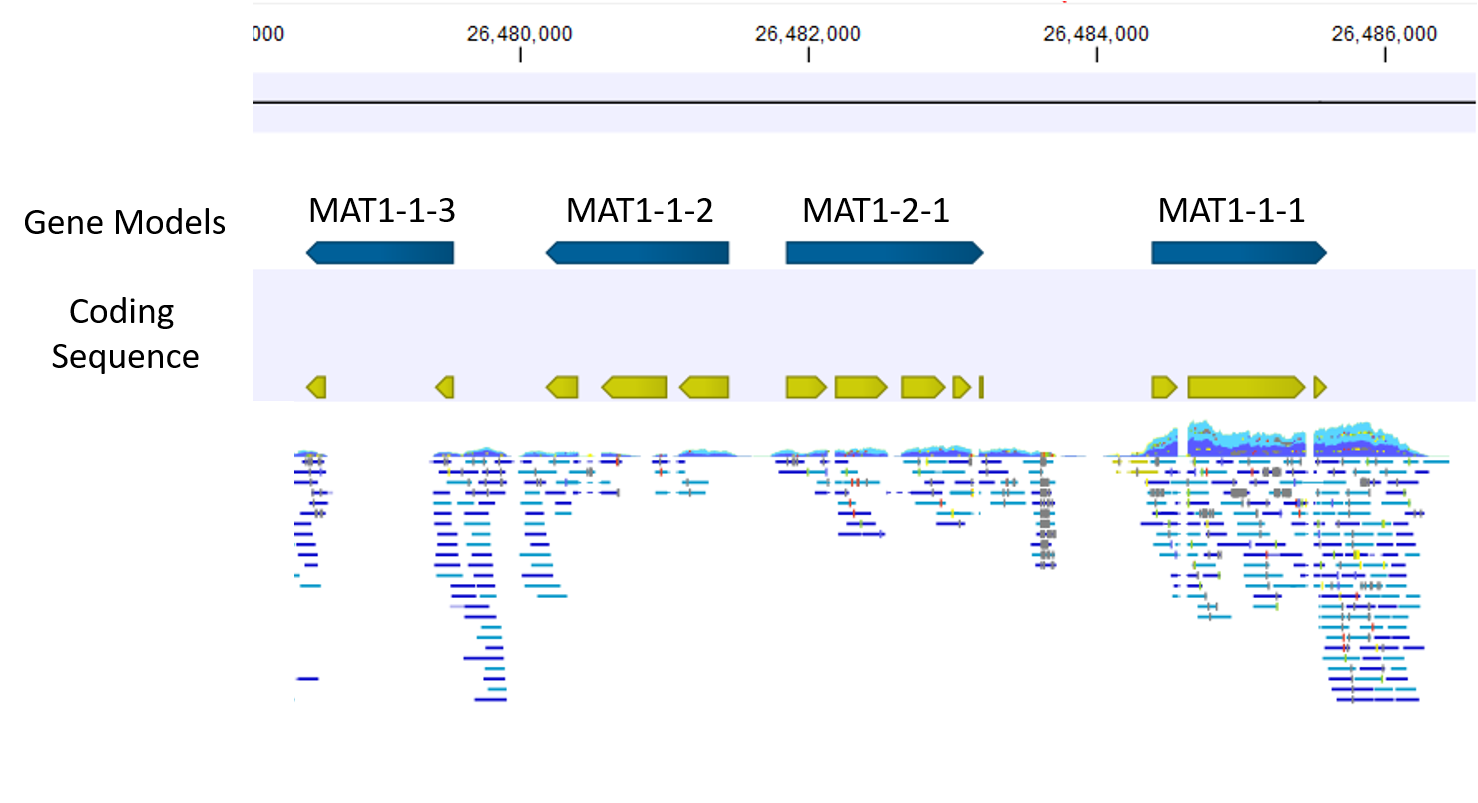
**

**Figure S3:** Mating type locus identified in *A. anomala* with RNA-seq evidence

Supplement: Supplementary file 12 — Supplementary Material 12. [file 12864_2024_10198_MOESM12_ESM.docx]
