# Supplementary material for: The massive 340 megabase genome of Anisogramma anomala, a biotrophic ascomycete that causes eastern filbert blight of hazelnut
Source: BMC Genomics. 2024 Apr 5;25:347. doi: 10.1186/s12864-024-10198-1 (PMC10998396; doi:10.1186/s12864-024-10198-1)
Supplement: Supplementary file 13 — Supplementary Material 13. [file 12864_2024_10198_MOESM13_ESM.docx]

**
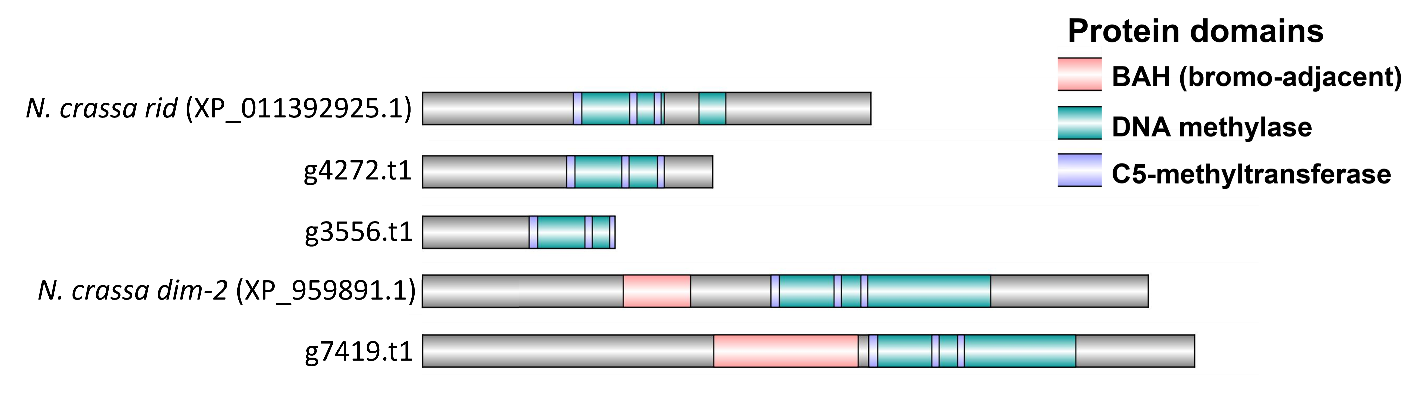
Figure S4:** Methyltransferases involved in the RIP process identified in *A. anomala* compared to their homologs in *N. crassa.*
